# Is “Physiological Lysis” in Viscoelastometry a Plasmin-Mediated Process?

**DOI:** 10.3390/diagnostics16101472

**Published:** 2026-05-12

**Authors:** Anikó Smudla, Herbert Schöchl, Andreas Calatzis, Csikós Richárd Gergely, János Fazakas

**Affiliations:** 1 Department of Intensive Therapy, Semmelweis University, 1082 Budapest, Hungary; smudla.aniko@semmelweis.hu (A.S.); fazakas.janos@semmelweis.hu (J.F.); 2 Ludwig-Boltzmann-Institute for Traumatology, The Research Centre in Cooperation with AUVA, 1200 Vienna, Austria; 3 Institute for Cardiovascular Prevention, LMU Munich University Hospital, 80336 Munich, Germany; andreas@calatzis.net; 4 Heart and Vascular Center, Semmelweis University, 1122 Budapest, Hungary; csikos.gergely@semmelweis.hu

**Keywords:** physiologic lysis, viscoelastometry, maximum lysis, tranexamic acid, clot retraction

## Abstract

Viscoelastic testing (VET) is widely used to guide hemostatic therapy in patients with coagulopathy. One important application is the detection of fibrinolysis, defined as a reduction in clot amplitude after maximum clot firmness (MCF), quantified as maximum lysis (ML). Low ML values have recently been associated with adverse outcomes in trauma and sepsis. However, the biological basis of low ML remains unclear. **Objective**: To determine whether low ML values reflect reduced plasmin-mediated fibrinolysis in tissue factor (TF) activated viscoelastic assays (EX-assay). **Methods**: A total of 120 healthy adults (52.5% female; mean age 38.2 ± 14.1 years) were studied. EX-assay without fibrinolysis inhibition were compared with assays containing the antifibrinolytic agent tranexamic acid (AP-assay). VET parameters obtained with and without fibrinolysis inhibition were compared using paired analyses, Pearson correlation, and Bland–Altman methods. **Results**: Clot firmness at 10 min (CA10) was similar with or without fibrinolysis inhibition; although MCF differed statistically, the difference was clinically negligible. ML ranged from 1% to 13% in both assays, with nearly identical mean values (5.9 ± 2.6% vs. 6.0 ± 2.6%). Correlation analysis demonstrated strong agreement for CA10, MCF, and ML between assays, and Bland–Altman analysis confirmed minimal bias for ML. **Conclusions**: Low ML values in TF-triggered viscoelastic assays were unaffected by tranexamic acid, suggesting that they are unlikely to reflect plasmin-mediated fibrinolysis. These findings support the contribution of alternative mechanisms, such as platelet-mediated clot retraction.

## 1. Introduction

Viscoelastic tests (VETs) are whole-blood coagulation assays used to evaluate clot formation, clot strength, and fibrinolysis. By integrating both cellular and plasmatic components of coagulation, VETs provide a comprehensive assessment of hemostasis. This methodology captures both chronometric parameters (such as clotting time and clot formation time) and structural parameters (such as measures of clot firmness), offering insight into the dynamics and quality of blood clot development [1,2]. Another advantage of VET is that it does not require blood centrifugation, allowing analysis to be performed directly on whole-blood samples [3,4,5]. As a result, VET provides rapid turnaround times, making it particularly valuable in acute clinical settings where timely assessment of coagulation status is essential [2,6].

Therefore, VETs are widely used to guide individualized hemostatic therapy in bleeding patients [7,8,9]. An important application is the detection of fibrinolysis, defined in VETs as a decline in clot amplitude after maximum clot firmness (MCF) has been reached [10,11,12]. Fibrinolysis exists on a spectrum from physiological fibrinolysis to hyperfibrinolysis and fibrinolytic shutdown [11,13,14].

Physiological fibrinolysis maintains vascular patency while preserving hemostasis. Normal reference ranges are device-specific and typically include: ROTEM^®^ EXTEM and ClotPro^®^ EX-test ML between 3 and 15%, TEG LY30 ranging 0.8–3%, and Quantra clot stability (Clot Stability to Lysis, CSL) > 90% [9,15]. Values within these ranges are considered physiologic and suggestive of a low-to-moderate fibrinolytic activity.

Hyperfibrinolysis (HF), by contrast, is characterized by excessive plasmin-mediated fibrin degradation and is defined by thresholds such as ML > 15% in ROTEM or ClotPro, LY30 > 7.5% in TEG, or >20% reduction in CSL in Quantra [9,16]. Excessive HF can result in rapid dissolution of fibrin clots and life-threatening hemorrhage [17,18,19]. In trauma, HF may be caused by tissue plasminogen activator (tPA) release, reduced plasminogen activator inhibitor-1 (PAI-1) activity, and activation of protein C [20,21,22], leading to uncontrolled plasmin activity, rapid clot breakdown, and high mortality [17,18,19,23].

At the opposite extreme, an absence of clot firmness reduction during the VET analysis (i.e., a low ML) has been termed “fibrinolysis shutdown” or “hypofibrinolysis” [24]. Definitions vary, but “fibrinolysis shutdown” is commonly defined as ML < 3% in ROTEM or ClotPro, LY30 < 0.8% in TEG, or no measurable CSL in Quantra [9]. “Fibrinolysis shutdown” has been reported in trauma, sepsis, and other critical illness, and is also associated with increased mortality [23,25,26].

The interpretation that low ML indicates a low “fibrinolysis” (which is commonly associated with a reduced plasmin activity) is widely accepted but has not been rigorously validated. In order to elucidate the mechanism of a low ML in the EX-assay, we compared assays that included potent pharmacological inhibition of fibrinolysis using tranexamic acid (AP-assay) with those that did not (EX-assay).

## 2. Materials and Methods

### 2.1. Study Design and Participants

Following approval by the local ethics committee (BM/22426-1/2024), the study was conducted at Semmelweis University Hospital, Budapest, in accordance with the Declaration of Helsinki. Written informed consent was obtained from all participants.

Eligible participants were healthy adults without anticoagulant therapy, medications influencing coagulation, or known hereditary bleeding disorders. Exclusion criteria were age < 18 years, pregnancy, anticoagulant intake, or hereditary coagulation disorders.

### 2.2. Blood Sampling and Laboratory Testing

After brief venous stasis, blood was collected into two 3.5 mL citrate tubes (3.2% trisodium citrate, VACUETTE^®^, Greiner AG, Kremsmünster, Austria; 1:9 citrate-to-blood ratio) and one 1.6 mL ethylenediaminetetraacetic acid (EDTA) tube for complete blood cell count. Samples were processed within 2 h from blood collection.

Routine coagulation assays included prothrombin time/international normalized ratio (INR), activated partial thromboplastin time (aPTT), and fibrinogen concentration (CoagXL analyzer, Diagon Kft, Budapest, Hungary). Complete blood cell count was performed using a Sysmex XN-1000 (Sysmex Corp., Kobe, Japan).

### 2.3. Viscoelastometry Analyses

**Figure 1:**
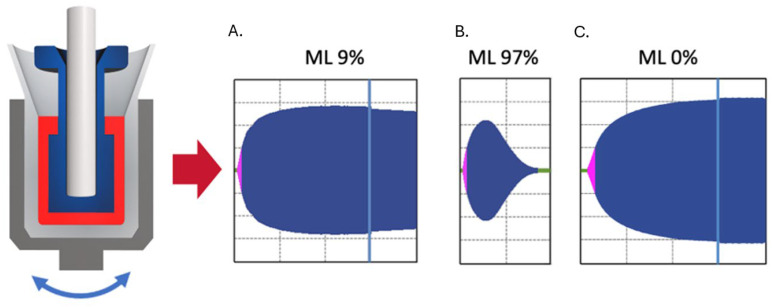
Principle of viscoelastometry on the ClotPro^®^ device and fibrinolysis phenotypes: (**A**). Physiological fibrinolysis, showing maximum lysis (ML) of 9% within the reference range. (**B**). Hyperfibrinolysis, characterized by complete clot dissolution. (**C**). Fibrinolysis shutdown, with no reduction in clot firmness after maximum clot firmness (MCF) is reached (ML = 0%). In the VET tracings shown, the first 30 min of the assay are displayed to the left of the blue line, while the final 10 min are shown to the right. This layout reflects the standard display format of the VET analyzer used in the study.

Viscoelastic testing was performed on the ClotPro^®^ analyzer (Diacare, Telki, Hungary) within 10 min of blood collection, following manufacturer instructions. The ClotPro system continuously detects the clot firmness by placing the blood sample between a cylindrical cup and a cylindrical pin. The cup is periodically rotated to the right and left using an elastic element (elastic motion thrombelastometry). Clot development is displayed as a curve, from which the clotting time (CT), clot amplitudes (CA5, CA10, CA20), maximum clot firmness (MCF), and maximum lysis (ML) are derived. ML quantifies the relative decline in clot firmness and is conventionally interpreted as a marker of fibrinolysis. For illustration, Figure 1 depicts physiological fibrinolysis, hyperfibrinolysis, and fibrinolytic shutdown patterns.

### 2.4. Assays

Two tissue factor (TF)-triggered viscoelastometry assays (Apiro Diagnostics, Budaörs, Hungary) were performed: **EX-assay:** TF-triggered viscoelastometry without fibrinolysis inhibition. The reagent contains calcium chloride (for recalcification), recombinant tissue factor, and polybrene (a heparin inhibitor). **AP-assay:** TF-triggered viscoelastometry with fibrinolysis inhibition, using tranexamic acid at a final concentration of 32 μg/mL, sufficient to fully block fibrinolysis in vitro. The AP-assay also contains calcium chloride and polybrene.

**Figure 2:**
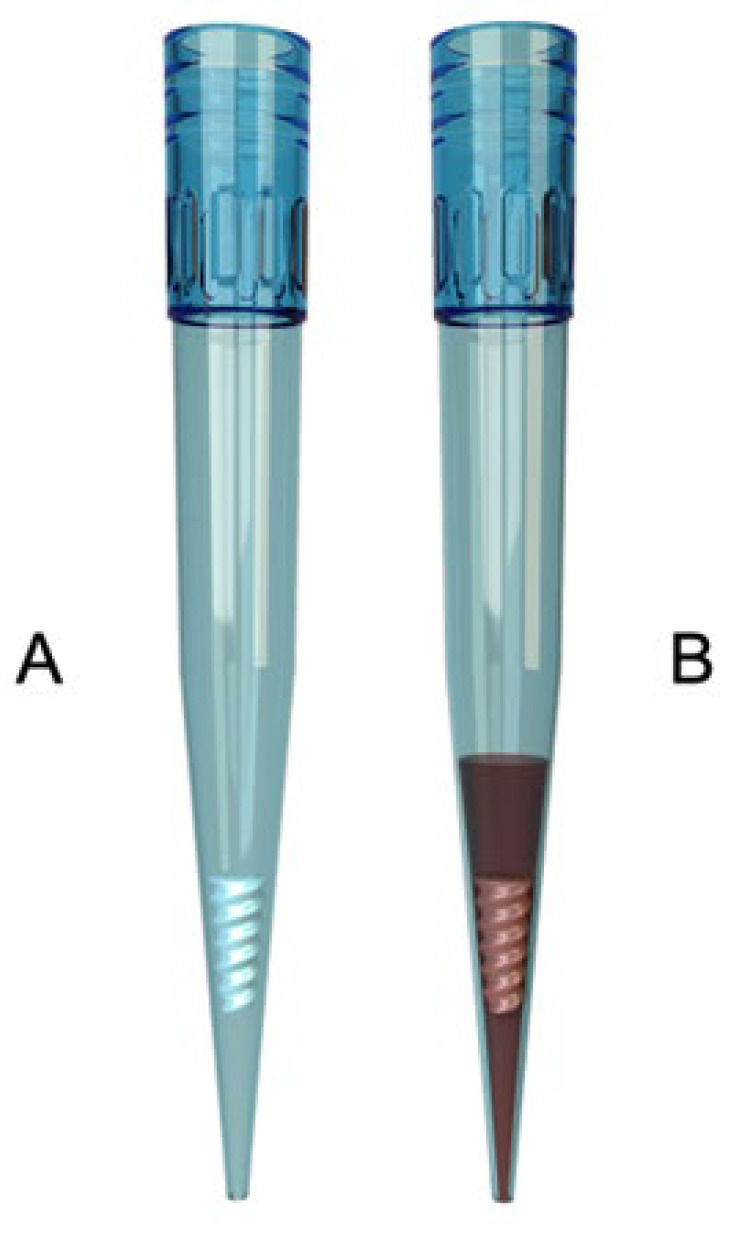
Apiro reagent tip. (**A**). The tip contains the test reagents in dry form, along with a flow modulator that ensures proper reconstitution. (**B**). Upon pipetting, the reagents are reconstituted directly into the blood. Graphic shown with permission from Apiro Diagnostics Kft.

Both assays were performed using Apiro reagent tips, which contain dry reagents and a flow modulator that ensures standardized reconstitution during pipetting of the whole-blood sample (Figure 2).

Measurements were performed at 37 °C for 60 min. The following parameters were recorded for both assays: CT, CA10, MCF, and ML. Clotting time (CT) refers to the time in seconds from test initiation to the first detectable clot formation (2 mm). The CA10 is the clot amplitude recorded at 10 min after the CT. Maximum clot firmness (MCF) represents the peak mechanical strength of the clot. Maximum lysis (ML), expressed as a percentage at the end of the measurement and represents the proportion of clot firmness that is lost after the MCF has been recorded. Reference ranges of the Apiro reagents are outlined in Table 1.

### 2.5. Statistical Analysis

Data distribution was assessed using the D’Agostino–Pearson test. Unless otherwise specified, results are presented as mean (standard deviation) or median and interquartile range (IQR). Differences between tests were analyzed by applying paired *t*-tests.

Correlations between viscoelastic parameters were determined using the Pearson correlation coefficient, with correlation strength classified as weak (0.30–0.49), moderate (0.50–0.69), strong (0.70–0.89), or very strong (0.90–1.00).

Agreement between the two devices was analyzed using Bland–Altman plots. For each viscoelastic parameter, the difference between paired measurements was plotted against their mean. The average bias, 95% limits of agreement, and the standard deviation (SD) of the bias were calculated.

A *p*-value < 0.05 was considered statistically significant. Statistical analysis and graphical representation were performed using Prism version 10.3.0 (GraphPad Software, Version 10.6.1., San Diego, CA, USA).

## 3. Results

A total of 120 healthy adults were enrolled, of whom 52.5% were female. The mean age was 38.2 ± 14.1 years (range, 19–79 years). Baseline hematological and coagulation parameters are presented in Table 2. Full blood counts and standard coagulation tests were within normal reference ranges.

### 3.1. Effect of Fibrinolysis Inhibition on Clotting Time and Clot Firmness Parameters (A10, MCF)

CT was significantly longer in the EX-assay compared with the AP-assay (*p* < 0.0001. On average, CT in the EX-assay exceeded the AP-assay by 9.9 s, reflecting the ClotPro software algorithm that automatically adds 10 s to the EX-test.

**Figure 3:**
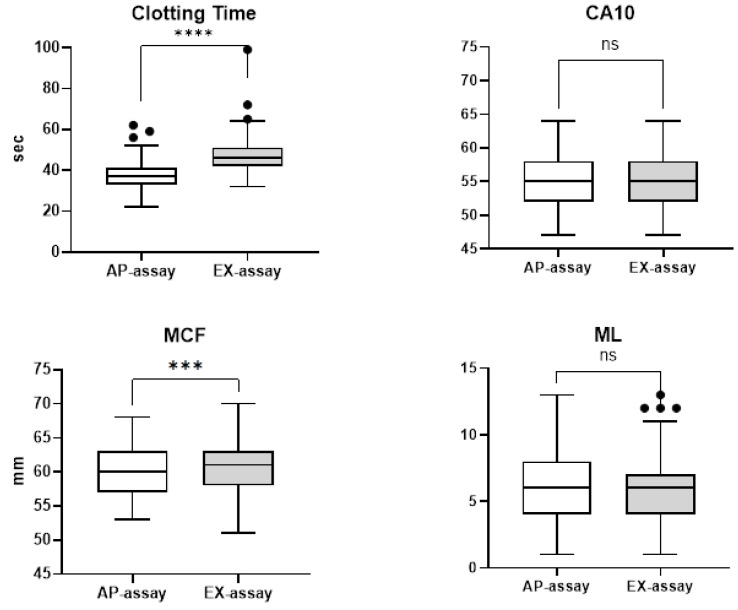
Differences between AP-assay and EX-assay. CA10, clotting time after 10 min running time; MCF, maximum clot firmness; ML, maximum lysis. Data are shown as box-and-whisker plots (median, interquartile range, and outliers). Paired *t*-tests. Significant differences are indicated as *** *p* < 0.001, **** *p* < 0.0001, ns, not significant.

In contrast, CA10 showed no significant differences between assays (Figure 3). A small but statistically significant difference was observed in MCF: 60.4 ± 3.7 mm in the AP-assay versus 60.2 ± 3.7 mm in the EX-assay (*p* < 0.001). Given the small difference (0.2 mm), this finding was considered clinically negligible.

### 3.2. Effect of Fibrinolysis Inhibition on Maximum Lysis (ML)

In the EX-assay, ML ranged from 1% to 13%, with a mean of 5.9 ± 2.6%. An ML above the reference range (ML > 12%) was observed in a single subject, while three participants (2.5%) demonstrated fibrinolysis below the reference range (ML < 2%). With potent fibrinolysis inhibition using tranexamic acid (AP-assay), ML values ranged from 1% to 13%, with a mean of 6.0 ± 2.6% (Figure 1).

### 3.3. Correlation Between EX- and AP-Assays

**Figure 4:**
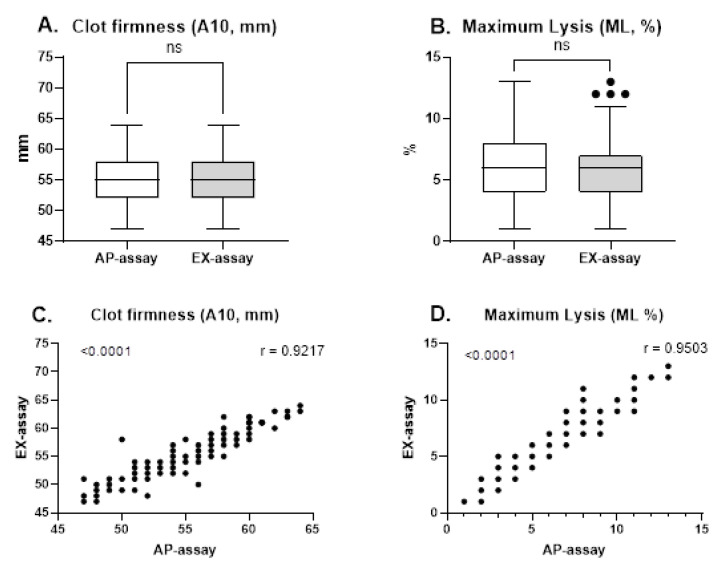
(**A**) Comparison of clot firmness (A10, mm) between AP-assay and EX-assay. (**B**) Comparison of maximum lysis (ML, %) between AP-assay and EX-assay. (**C**) Pearson correlation analysis of clot firmness (A10, mm) between AP-assay and EX-assay. (**D**) Pearson correlation analysis of maximum lysis (ML, %) between AP-assay and EX-assay. A10, clot firmness after 10 min running time; ML, maximum lysis.

As shown in Figure 4, CA10 and ML values obtained with EX-assay and AP-assays were closely correlated. In contrast, the correlation between AP-assay and EX-assay for CT was only moderate. Regression analysis yielded a slope approaching unity, indicating no systematic difference.

### 3.4. Agreement Between EX-Assay and AP-Assay

**Figure 5:**
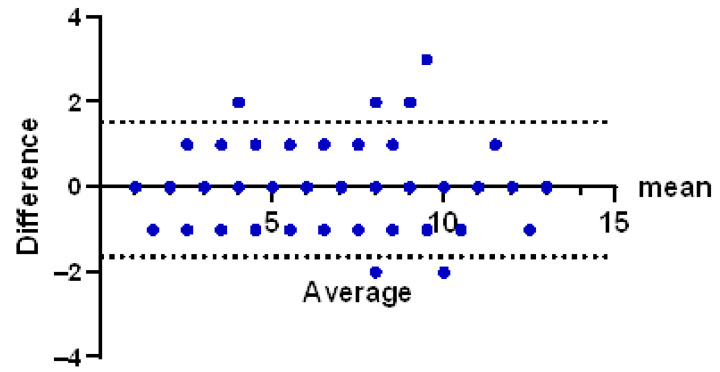
Bland–Altman plot of maximum lysis values obtained with and without fibrinolysis inhibition.

The Bland–Altman plot demonstrated a mean bias close to zero with narrow limits of agreement, suggesting that any differences between assays are negligible and not clinically relevant (Figure 5).

## 4. Discussion

This study demonstrates that in healthy individuals, recorded maximum lysis levels (ML ≤ 13%) in tissue factor-triggered viscoelastometry (EX-assay) is unaffected by complete pharmacological inhibition of fibrinolysis (AP-assay). In both conditions, without and with tranexamic acid (TXA), ML values ranged from 1 to 13% with nearly identical means (5.9 ± 2.6% vs. 6.0 ± 2.6%). Similarly, clot firmness parameters (CA10, MCF) were unchanged irrespective of fibrinolysis inhibition. These findings show that the modest decline in clot firmness observed in the reference range of the ML parameter does not reflect plasmin-mediated fibrin degradation.

### 4.1. Fibrinolysis and Viscoelastometry

Fibrinolysis is the enzymatic degradation of fibrin by plasmin and represents a crucial mechanism for maintaining blood vessel patency and prevention of thrombosis [27]. In viscoelastometry, fibrinolysis appears as a progressive reduction in clot firmness after the MCF has been recorded, quantified as ML or lysis indices (LI, LY). Experimental data indicate that viscoelastometric evidence of fibrinolysis requires marked biochemical activation. Raza et al. reported detectable lysis in trauma patients only when plasmin–antiplasmin (PAP) complexes were >30-fold above baseline and antiplasmin activity < 75% of normal [28].

Hyperfibrinolysis, typically defined as ML > 15% in TF-triggered assays without exogenous tPA, represents excessive plasmin activation and rapid clot dissolution [12,17,19]. It is most often encountered in trauma, major surgery, cardiopulmonary resuscitation, or postpartum hemorrhage [18,29,30,31], usually driven by surges of endothelial tPA during shock [32]. Hyperfibrinolysis carries a high mortality risk if untreated, but the use of TXA in trauma protocols has markedly reduced its incidence [33].

Low ML values (<15%) have been widely labeled as “physiologic” lysis [13]. Our data, however, demonstrate that such values persist despite potent fibrinolysis inhibition, arguing against plasmin as the underlying mechanism.

### 4.2. Clot Retraction Versus Fibrinolysis

A plausible explanation for low ML is clot retraction, a platelet-driven process in which activated platelets contract the fibrin network, compact the clot, and expel serum [34]. Although clot retraction cannot physically draw the viscoelastometer’s pin and cup together, the contractile forces may subtly alter the recorded amplitude. In healthy blood, this produces a modest decline in clot firmness independent of fibrinolysis. In trauma, sepsis, or surgery, impaired platelet contractility could blunt this effect, resulting in low ML values that may be misclassified as “shutdown”.

This concept is not new. In his 1948 description of thromboelastography, Hellmut Hartert attributed the late phase of the curve partly to clot retraction [35]. In a later TEG study, Takeshi Katori and colleagues used platelet-poor plasma (PPP) and platelet-rich plasma (PRP) with varying platelet counts and found that higher platelet counts led to a greater decrease in amplitude after maximum amplitude (MA). Furthermore, D-dimer levels were not elevated. These findings demonstrate that the observed decrease in clot amplitude was caused by platelet-mediated clot retraction rather than fibrinolysis [36].

Newer viscoelastometry protocols, however, interpret such late amplitude declines as “physiologic fibrinolysis”, and the absence of such declines as a “fibrinolysis shutdown”. Our results suggest that ML in the physiological range (1–13%) likely reflects clot retraction or other plasmin-independent mechanisms.

### 4.3. Mechanistic Implications

The distinction between fibrinolysis and clot retraction has important mechanistic consequences. Fibrinolysis is a plasmatic process controlled by tPA, PAI-1, plasminogen, and α2-antiplasmin [27]. By contrast, clot retraction is a cellular function dependent on platelet actomyosin contraction and integrin signaling [37]. Thus, the same viscoelastometry parameters (ML, LY30, LI60) may reflect entirely different biological pathways. This methodological limitation of viscoelastometry should be acknowledged.

### 4.4. Clinical Implications

Equating low ML with fibrinolysis shutdown risks clinical misinterpretation. Trauma patients with ML < 3% are often categorized as shutdown and considered at increased risk of mortality [13,23]. Yet, if these values instead reflect impaired clot retraction, treatment decisions may be misguided. Withholding antifibrinolytics based on a presumed shutdown could deny patients beneficial therapy, while unnecessary interventions could be pursued to target a nonexistent suppression of fibrinolysis [23].

In research, reliance on ML thresholds may bias estimates of fibrinolytic phenotypes and their associations with outcomes. The correlation between low ML/LY30 and mortality may persist but likely reflects platelet dysfunction rather than impaired fibrinolysis. Platelet contractility itself contributes to clot stability, thrombus remodeling, and wound healing [32]. If low ML reflects impaired platelet contractility, this could represent a clinically relevant mechanism of hemostatic dysfunction with prognostic implications.

### 4.5. Alternatives to TF-Triggered Assays

A more reliable method for assessing antifibrinolytic capacity is the tPA-challenge assay (TPA-assay), in which exogenous tPA is added in vitro to probe plasmin generation. These assays are highly sensitive to TXA inhibition [38,39,40] and can reveal impaired fibrinolytic responsiveness in critically ill patients, including trauma and sepsis [41,42]. By directly testing fibrinolytic response to tPA, the tPA-challenge can determine a true fibrinolytic shutdown.

Clinical studies evaluating tPA-challenge have already shown value in stratifying fibrinolytic phenotypes and predicting transfusion needs in trauma, sepsis, and postpartum hemorrhage [26,42,43,44,45,46,47]. Our findings support incorporating such assays into both research and clinical practice, rather than relying solely on low ML values in TF-triggered assays.

### 4.6. Limitations

This study has limitations. First, only healthy volunteers were studied; results may differ in patients with coagulopathy. Therefore, it would be desirable that future studies evaluate our findings in critically ill or bleeding patients. Second, analyses were restricted to the ClotPro platform. While comparable ML ranges have been reported on ClotPro and ROTEM, it would be valuable for future studies to investigate “physiological fibrinolysis” both in the presence and absence of pharmacological inhibition of plasmin-mediated fibrinolysis using TEG, ROTEM, Quantra or MultiClot analyzers. Another limitation is that only tissue factor (TF)-triggered VET was evaluated, whereas native VET assays (e.g., NaTEM) and assays activated by ellagic acid (e.g., InTEM) or kaolin (e.g., CK on TEG) were not assessed. However, given the high concentration of tranexamic acid tested (32 µg/mL), this is unlikely to have influenced its inhibitory effect on plasmin-mediated fibrinolysis. Consistent with this, a review by Picetti reported that the applied TXA concentration is sufficient to reliably suppress fibrinolysis regardless of the activation pathway used to generate thrombin [48]. Finally, we did not perform direct measurements of clot retraction or biochemical fibrinolytic markers (PAP complexes, D-dimer, PAI-1), so the role of retraction remains inferential rather than directly demonstrated.

Second, analyses were restricted to the ClotPro platform, although comparable ML ranges have been reported on ROTEM. Third, we did not perform direct measurements of clot retraction or biochemical fibrinolytic markers (PAP complexes, D-dimer, PAI-1), so the role of retraction remains inferential.

## 5. Conclusions

In summary, in our study, low ML values (≤13%) in TF-triggered viscoelastometry were unaffected by complete pharmacological inhibition of fibrinolysis. Our data, therefore, suggests that low ML does not reflect plasmin-mediated fibrin degradation, and ML values below the reference range should not be equated with fibrinolysis shutdown. Alternative mechanisms, particularly platelet-driven clot retraction, likely account for the modest amplitude decline recorded in viscoelastometry in samples from healthy individuals. Diagnosis of a “fibrinolysis shutdown” should rather be performed using assays that directly assess fibrinolytic response, such as the tPA-challenge. Clinicians and researchers should interpret low ML values with caution to avoid misclassification and mismanagement.

## Figures and Tables

**Table 1:** Reference ranges of the Apiro reagents. CA 10, clot amplitude 10 min after CT; MCF, maximum clot firmness; ML, maximum lysis. EX-assay, extrinsically active test; AP-assay, extrinsically active test plus tranexamic acid.

	Clotting Time (s)	CA 10 (mm)	MCF (mm)	ML (%)
EX-assay	37–65	48–63	53–67	2–12
AP-assay	28–56	47–63	54–67	2–11

**Table 2:** Full blood count and routine coagulation tests. SD: standard deviation, IQR: interquartile range, WBC, white blood cell count; Hct, hematocrit; INR, international normalized ratio; aPTT, activated partial thromboplastin time; Fg, fibrinogen.

	WBC (G/L)	Hct (%)	Platelet Count (G/L)	INR	aPTT (s)	Fg (g/L)
mean ± SD	6.7 ± 1.6	42.3 ± 4	270 ± 64	1.01 ± 0.07	28.9 ± 2.7	3.0 ± 0.6
min–max	2.9–11.7	34–53	112–454	0.9–1.2	22.4–37.3	1.1–4.6
IQR	5.7–7.7	40–45	229–315	1–1.1	27.1–30.5	2.5–3.3

## Data Availability

The data presented in this study are available on request from the corresponding author.

## Abbreviations

The following abbreviations are used in this manuscript:

AP-assay	Extrinsically activated assay plus tranexamic acid
apTT	activated partial thromboplastin time (aPTT)
CA 5/10	Clot amplitude after 5 or 10 min running time
CSL	Clot Stability to Lysis
CT	Clotting time
EX-assay	Extrinsically activated assay
Hct	Hematocrit
HF	Hyperfibrinolysis
INR	international normalized ratio
LI	Lysis index
LY	Clot lysis
MCF	Clot firmness
ML	Maximum Lysis
PAI-1	plasminogen activator inhibitor-1
PAP	Plasmin–Antiplasmin
ROTEM	Rotational Thrombelastometry
TEG	Thromboelastography
TF	tissue factor
tPA	Tissue plasminogen activator
TXA	Tranexamic acid
VET	Viscoelastic test
WBC	white blood cell count
